# Editorial: Discovery of small molecule lead compounds: a driving force to unravel new anti-cancer targets and mechanisms, Volume II

**DOI:** 10.3389/fphar.2024.1519858

**Published:** 2024-12-18

**Authors:** Yan Zhang, Leilei Fu, Muhammad Umer Farooq Awan, Yinu Wang

**Affiliations:** ^1^ School of Traditional Chinese Materia Medica, Shenyang Pharmaceutical University, Shenyang, China; ^2^ School of Life Science and Engineering, Southwest Jiaotong University, Chengdu, China; ^3^ Department of Botany, Government College University, Lahore, Pakistan; ^4^ Feinberg School of Medicine, Northwestern University, Chicago, United States

**Keywords:** anticancer natural products, leading compound, high-throughput screening, potential cancer targets, target recognition technology

Natural products with complex structures and diverse biological activities are important sources of new anticancer drugs, with the ability to reverse multi-drug resistance. The search for lead compounds with new molecular targets, such as enzymes, receptors, and genes, is also an effective way to find new anticancer drugs. Computational biology and high-throughput screening can additionally be helpful in the discovery of drug candidates. This Research Topic aims to explore the latest developments in this field and consists of seven articles. We have had the privilege of reviewing a wide range of research articles and reviews in the field. Here, we summarize the main findings and ideas detailed in each of the accepted articles.

Akash et al. used computational method to evaluate colorectal cancer inhibitors of resveratrol derivatives, and selected the top seven compounds according to their scores. Then, the Lipinski Rule, pharmacokinetics, ADMET profile study, molecular orbital analysis, molecular docking, molecular dynamics simulation and MM-PBSA combined free energy calculation were used to analyze the drug and target interaction. The compounds [(03) Resveratrol 3-beta-mono-D-glucoside, and (29) Resveratrol 3-Glucoside] showed the highest levels of effectiveness, and all of the molecules were non-toxic. Molecular dynamics simulations were then performed again to confirm the stability of the first two ligand-protein complexes. The results show that resveratrol derivatives may be effective candidates for the control of colorectal cancer and have important research value.

In this study, Li et al. designed and synthesized 94 lead compounds to investigate the inhibitory effect of this series of compounds on BRD4. The results showed that DDT26 had the strongest inhibitory effect on BRD4, and showed significant anti-proliferation activity on both TNBC cell line and MCF-7 cell line. Further experiments showed that DDT26 showed a moderate inhibitory effect on PARP1, and could also regulate c-MYC and *γ*-H2AX induced DNA damage, inhibit cell migration and colony, and block the cell cycle in the G1 phase in MCF-7 cells. The results of this study provide candidate drugs for anti-breast cancer agents targeting BRD4, and also indicate that BRD4 inhibition has shown good potential in the treatment of TNBC.

Atanasova et al. developed a new morphology-based screening method using organoids to screen 144 compounds in a compound library and found that 9 of them showed better effects. The ADM reversal effect of the priority compounds was validated in p48Cre/+ and found that the molecular index analysis of ADM reversal in KC mouse organoids is improved compared with TSA. Meanwhile, RNA sequencing shows that angiotensinogen is the most inhibited pathway in the process of ADM reversal. This discovery reveals a unique epigenetic mechanism, suggesting that the phenotypic screening method established in this study can be applied to the discovery of potential treatments for PDAC.

There has been considerable interest in small molecule inhibitors of HK2 in the field of cancer therapy, but the development of selective inhibitors of HK2 remains a gap. Chakraborty et al. proposed a multi-target modeling approach to design a series of HK2 ligands with anti-proliferative activity against FaDu and Cal27 oral cancer cell lines. They also proposed an environmentally friendly one-step synthesis process to synthesizing the lead compound H2, which has cell cycle arrest and apoptosis-inducing effects, as well as reducing the growth of tumor spheroids.

Multidrug resistance is the biggest challenge in cancer treatment worldwide, which often leads to the failure of clinical chemotherapy. Multiple studies have shown that natural lead compounds can reverse multidrug resistance, and this line of research has become a hot area of cancer research. Studies have shown that natural products such as flavonoids, alkaloids, terpenoids, polyphenols and coumarins can reverse multidrug resistance by regulating signaling pathways or related protein expression. Zou et al. systematically summarized the progress of natural products in reversing multidrug resistance, providing some references for the study of multidrug resistance.

Liu et al. demonstrated that Shikonin can promote the apoptosis of colon cancer cell lines HT29 and HCT116, and has antibacterial effects. Sa-*β*-galactosidase staining also showed that the cell senescence rate of colon cancer cells was significantly increased after treatment with Shikonin. The results show that Shikonin has dual anti-aging and anti-tumor effects. The study provides new molecular mechanisms and potential therapeutic targets for the treatment of colon cancer.

The research on novel PHGDH inhibitors with high efficiency, low toxicity, and diverse structures has become a strategy for drug discovery. Xu et al.established a 3D-QSAR ligand-based pharmacophore model using the HypoGen algorithm in Discovery Studio. Through the established algorithm, they screened potential PHGDH inhibitors. Ultimately, they successfully identified three potential anti-cancer candidate drugs.

